# Strength training improves heart function, collagen and strength in rats with heart failure

**DOI:** 10.1186/s12576-024-00899-3

**Published:** 2024-02-16

**Authors:** Leisiane G. Dias, Carlos H. O. Reis, Leonardo dos Santos, Walter Krause Neto, Ana Paula Lima-Leopoldo, Julien S. Baker, André S. Leopoldo, Danilo S. Bocalini

**Affiliations:** 1 Experimental Physiology and Biochemistry Laboratory. Physical Education and Sport Center, Federal University of Espirito Santo, Vitoria, Brazil; 2 Department of Physiological Sciences, Health Sciences Center, Federal University of Espirito Santo, Vitoria, Brazil; 3 Department of Morphology and Genetics, Federal University of São Paulo, São Paulo, Brazil; 4 Department of Sport, Physical Education and Health, Centre for Health and Exercise Science Research, Hong Kong Baptist University, Kowloon Tong, Hong Kong

**Keywords:** Exercise, Myocardial remodeling, Contractility, Resistance training, Myocardial fibrosis

## Abstract

**Background/objectives:**

Myocardial infarction (MI) frequently leads to cardiac remodeling and failure with impaired life quality, playing an important role in cardiovascular deaths. Although physical exercise is a well-recognized effective non-pharmacological therapy for cardiovascular diseases, the effects of strength training (ST) on the structural and functional aspects of cardiac remodeling need to be further documented. In this study, we aimed to investigate the role of a linear block ST protocol in the rat model of MI.

**Methods and results:**

After 6 weeks of MI induction or sham surgery, male adult rats performed ST for the following 12 weeks. The ladder-based ST program was organized in three mesocycles of 4 weeks, with one load increment for each block according to the maximal carrying load test. After 12 weeks, the infarcted-trained rats exhibited an increase in performance, associated with reduced cardiac hypertrophy and pulmonary congestion compared with the untrained group. Despite not changing MI size, the ST program partially prevented cardiac dilatation and ventricular dysfunction assessed by echocardiography and hemodynamics, and interstitial fibrosis evaluated by histology. In addition, isolated cardiac muscles from infarcted-trained rats had improved contractility parameters in a steady state, and in response to calcium or stimuli pauses.

**Conclusions:**

The ST in infarcted rats increased the capacity to carry mass, associated with attenuation of cardiac remodeling and pulmonary congestion with improving cardiac function that could be attributed, at least in part, to the improvement of myocardial contractility.

**Supplementary Information:**

The online version contains supplementary material available at 10.1186/s12576-024-00899-3.

## Introduction

In 2019, almost 19 million deaths were attributed to cardiovascular diseases (CVD), which represented an increase of 17.1% compared to 2010 [1]. CVDs are a group of disorders where ischemic heart disease and myocardial infarctions (MI) are the most impactful and damaging. Despite the great advances in the cardiovascular therapies in the last decades, MI remains the most common cause of heart failure (HF) in those patients who survive acute insults. Following the ischemic event that culminates in myocardial necrosis, the consequent chamber overload induces remodeling and hypertrophy of the remnant myocardium, which imply in contractile impairment and intensify of global cardiac dysfunction characterized by exercise intolerance and a significant impairment on health-related quality of life [2].

It has also been documented that a sedentary lifestyle and physical inactivity have contributed to the increased incidence and prevalence of chronic illness such as CVD, and individuals need to implement strategies that modify these aspects of lifestyles. Involvement in physical activity and physical training have been widely investigated to reduce morbidity and mortality. Different aerobic training protocols (e.g., swimming and walking) have been shown to have positive effects on the cardiovascular system, both in humans and animals [3–9]. Specifically aiming the preservation of cardiac function post-MI, many animal studies on treadmill or swimming trainings have been extensively performed [10, 11, 11, 12, 12, 13, 13], demonstrating positive effects on cardiac remodeling [14], coronary perfusion [15, 16] and cardiomyocyte calcium handling [17].

In addition, strength training (ST) has also been shown to be effective in reversing skeletal muscle atrophy and improving quality of life [3, 18–22], enhancing VO_2_ max. [23], increasing strength [24] and muscular endurance [25] in patients post-MI. Studies indicate that isolated ST [26–29] or combined with aerobic exercises [23, 30, 31], are also beneficial in animal models of MI without adverse events [32–35]. These effects include improvement in VO_2_peak [27], metabolism and structure of skeletal muscle [28, 36], immune and autonomic modulation [29, 37] and increase in muscle strength [28, 38, 39]. However, the effects of ST on the structural and functional aspects of cardiac remodeling need further investigation and documentation. Thus, the aim of the present study was to investigate the role of a structured ST protocol on cardiac remodeling and myocardial function in rats submitted to an MI model.

## Materials and methods

### Animal care and study design

All protocols were previously evaluated and approved by the institutional Committee on ethics in the use of animals (project number 10/2021). Young (8–10 weeks old) disease-free male Wistar rats (*n* = 30) were provided by the UFES Central Animal Facility and kept in collective boxes with water and food “ad libitum”, in an environment with temperature control (22 ºC), air humidity (54%) and light cycle/dark (12/12 h). The animals were randomly distributed into controls (sham surgery) or infarcted (coronary occlusion). After 6 weeks, surviving infarcted animals were redistributed and kept untrained or trained for the following 12 weeks. For the final analysis, three experimental groups were composed: control (C; *n* = 7–10), infarcted (MI; *n* = 9–10), and infarcted trained (MIT, *n* = 8–10).

### Myocardial infarction induction

Experimental MI was produced according to a standardized model described by previous studies [40, 41]. Briefly, animals were anesthetized with a mixture of ketamine (50 mg/kg i.p.) and xylazine (10 mg/kg i.p.), and after orotracheal intubation (Gelko-14G), mechanical ventilation was instituted with a ventilator (Rodent ventilator Mod 683, Harvard Apparatus©, Boston-MA, USA) with a frequency of 90 movements/minute and a tidal volume of 2.0 ml. Through a left thoracotomy using the third intercostal space as a reference, the heart was exteriorized and the anterior interventricular branch of the left coronary artery was occluded at the level of the lower border of the left atrium with 6.0 mononylon thread, causing permanent arterial occlusion. Next, the heart was internalized and the thorax immediately closed using a purse-string ligature, previously prepared. Thereafter, animals were removed from artificial ventilation and natural breathing was stimulated. Because cardiac dysfunction becomes evident after the healing period of large MI, only animals with MI occupying more than 35% of the left ventricle (LV) as measured by echocardiography performed 48 h post-surgery were used. The control group consisted of animals submitted to sham coronary occlusion surgery (underwent thoracotomy without artery occlusion) and remained without physical training for the following 18 weeks.

### Echocardiography

After 48 h of surgery, the surviving animals supposedly infarcted were submitted to echocardiography to confirm MI by detecting and measuring the extent of MI in relation to the LV wall. Examination was done by one observer blinded to the animal group, with a HP SONOS 5500 (Philips Medical Systems, Andover, MA, USA) and a 12-MHz/2-cm transducer, according to previously detailed technique [40–42]. The infarct size was measured by echocardiography at basal, medium and apical LV levels, by analyzing the length of the arc corresponding to the hypokinetic and/or akinetic portion (infarcted area) and the total perimeter of the LV endocardial border at the end of diastole. Thus, the relative MI size was expressed in % of the LV averaged in three planes. This echocardiographic approach has previously been proven to be accurate in estimating infarct size compared to histochemical method [43]. Then, all infarcted rats remained at rest for six weeks for complete infarct healing. At the end of the sixth week, infarcted rats were subsequently randomized in MI or MIT groups and 12 weeks after training protocol a complete Doppler echocardiography was performed in all groups for the morphofunctional evaluation of the LV. The LV end-diastolic and end-systolic transverse areas were measured and fractional area shortening (FAS) was calculated. The parameters of mitral diastolic inflow velocity curves derived from pulsed-wave Doppler (peaks of E- and A-wave velocities) were used to assess diastolic function.

### Strength training program

The apparatus used to perform ladder-based resistance (strength) training was adapted from previous studies [44, 45] and consisted of a vertical ladder measuring 110 cm high by 18 cm wide, 80° inclination, and with spacing of 2 cm between steps. At the top there was a 20 × 20 × 20 cm chamber for rest between climbing sets [45], and at the base, an elevated structure was fixed avoiding any contact of the animal's tail or the load system with the ground. To fix the loads to the animal, stainless steel cable was used to attach cylinder weights.

Adaptive training and apparatus familiarization consisted of rats performing five attempts per day from different points on the ladder: near the top, in the middle, and the bottom; during ten consecutive days without any load attached to the animal’s tail. In the first week, the animals remained for 120 s in the rest chamber (at the top of the ladder) in order to realize that the environment did not present any danger to the animals. In the first and second attempts, animals were placed near to the top at a distance of approximately 35 cm from the chamber entrance. On the third and fourth attempts, the animals were positioned in the middle, and in the last attempt, the animals were placed at the bottom (beginning) of the ladder, 110 cm away from the chamber entrance.

Ten days after complete adaptation, the maximum carrying load capacity was assessed for each rat by performing climbs with progressively heavier loads. On the first attempt, rats climbed the ladder carrying a load equivalent to 70% of their body mass. Then, an additional 30 g of load was added at every successful climb until the rats could not climb the entire ladder for three consecutive attempts with a 5-min interval between attempts. The heaviest load successfully carried was considered the maximum carrying load. The maximum load was assessed every week for each rat. The ST protocol consisted of 12 daily climbs with different loads referring to the % of the maximum carrying load, with a 90-s recovery interval between sets, five times a week, for 12 weeks, as previously described in a study by our group [46]. The ladder length permits 8 to 12 movements (repetitions) to climb from the bottom to the top. The load evolutions are described in Table 1, being increased proportionally to the training period during the week period.

**Table 1:** Strength training sessions design

Week	Block 1				Block 2				Block 3			
	1	2	3	4	5	6	7	8	9	10	11	12
Intensity (%)	60	65	70	75	60	65	70	75	60	65	70	75

% intensity in relation to the maximum carrying load

### Hemodynamic assessment

Cardiac hemodynamics were assessed at the end of the 18th week of experiment with the animals anesthetized with urethane (1.2 g/kg, i.v., Sigma, MO, USA), kept warm (± 37 °C) and under mechanical ventilation (frequency: 100 movements/minute and tidal volume: 10 ml/kg) enriched with oxygen, according to the standardized technique in the laboratory [42]. The right femoral vein was catheterized to maintain the anesthetic plane, drug and hydro saline administration. Right ventricle (RV) catheterization was performed from the right external jugular vein through a PE20 polyethylene catheter 8 cm in length, whose distal end was positioned inside the RV. To assess LV pressures, a micromanometer (MikroTip® 2F, Millar Instruments Inc., Houston, TX, USA) had its distal end positioned inside this cavity after catheterization of the right common carotid artery. Intraventricular pressure data were obtained using AcqKnowledge® 3.7.5 software (Biopac Systems Inc., CA, USA) and the following parameters was measured or calculated: systolic and end-diastolic ventricular pressures (mmHg), heart rate (bpm) and first temporal derivative of pressure (dP/dt, in mmHg/s).

### Determination of cardiac masses and lung water content

The atrial and ventricular masses were measured, and lung water content was estimated as previously detailed [40, 41]. Briefly, the heart was dissected, separating the right and left atria, as well as the left and right ventricles, and all chambers were weighed on a calibrated precision scale. In addition, lung water content was estimated in a sensitive analysis and an indicator of tissue congestion, as verified in other studies [40, 41]. After the killing of the animals in the experimental groups, the right lung was isolated by stitching with cotton thread around the pulmonary hilum to avoid fluid loss during manipulation. It was then removed from the animal and immediately weighed, which allowed calculation of wet mass. Tissue samples were kept in an oven (85 ºC for 12 h) and later weighed. After that, the dry mass of the samples was determined. Once the dry mass of the lung was reached, the water content (%H_2_O) was defined by the equation:

$$\% {\text{H}}_{{2}} {\text{O }} = \, \left( {{\text{wet mass }}{-}{\text{ dry mass}}} \right) \, /{\text{ wet mass x 1}}00.$$

### Myocardial contractility assessed “in vitro”

Mechanical performance of the cardiac muscle was evaluated in papillary muscles isolated from the LV. Throughout a median thoracotomy, the hearts were quickly removed and placed in a Krebs–Henseleit nutrient solution (132 NaCl; 4.69 KCl; 1.5 CaCl; 1.5 MgSO_4_; 1.18 KH_2_PO_4_; 5.50 glucose and 20 of HEPES as a buffer) previously heated to 29 °C and bubbled with oxygen according to the standard technique [3, 10]. Briefly, the right ventricular free wall was excised to expose the interventricular septum, which was sectioned to access the LV papillary muscles. The posterior papillary muscle was carefully dissected, and its ends were fixed by stainless steel rings. Then, the muscles were transferred to a glass chamber filled with oxygenated Krebs–Henseleit nutrient solution kept at 29 °C, and were electrically stimulated (model AVS-100, AVS Projetos Especiais, São Paulo, SP, Brazil) by square waves with a duration of 5 ms and a voltage approximately 10–15% higher than the minimum necessary to provoke a mechanical response, at a frequency of 0.2 Hz. Then, the muscle was carefully stretched by micromanipulator (model 2046F, Mitutoyo Sul Americana, São Paulo, SP, Brazil) attached to force transducer (model FT03E, Grass Instruments, Quincy, MA, USA), until it reached the top of the length/tension curve (L_max_: diastolic length of the muscle in which the developed isometric tension reached the maximum value). The following parameters were analyzed in L_max_: developed tension (DT); rest tension (RT); rate of tension variation, considering the maximum positive (+dT/dt_max_) and negative (-dT/dt_max_) rates, time to peak of tension (TPT) and time for 50% relaxation (TR50%). After obtaining data under steady-state conditions, the following protocols were also performed:

Post-pause potentiation: Pauses of stimuli (5, 10, 15, 30, 45, 60 and 120 s) was produced, and the twitch that immediately followed the pause normalized by the contraction that preceded the pause was used to provide information on the calcium cycling [47–49].

Length–tension curves: After stabilization at L_max_ (100%), the DT was determined in the lengths corresponding to 92%, 94%, 96%, and 98% of L_max_, which enabled the determination of the active length–tension curve (Frank–Starling relationship) and passive length–tension curve (myocardial stiffness) [3, 10].

Contractile response to extracellular calcium: After stabilization the nutrient solution containing 1.5 mM of calcium was replaced by another containing a higher concentration of calcium (2.5 mM).

Once the maneuvers assessing myocardial values were completed, the muscles were removed from the system and the segment between the steel rings was isolated and weighed on an OCCU-124 model scale (Fisher Scientific, Pittsburg, PA, USA). Thereby, tension measured obtained in grams was then corrected by the respective cross-sectional area (g/mm^2^).

### Histopathological study

After weighing and transversely cut, the apical half of LV was fixed with 4% formalin buffered with phosphate buffer solution (0.01 mM; pH 7.4) to obtain sections cut from the mid-transversal face of the heart, for microscopy according to Antonio et al. [40]. The samples were processed in an auto-technical device with a total cycle of 12 h for dehydration, clearing and paraffinization. Dehydration was performed with increasing concentrations of ethyl alcohol until reaching absolute alcohol, cleared in xylol, impregnated and embedded in paraffin at 60ºC to obtain 3 μm cuts in a Minot-type microtome (Leica Microsystems Ltd, Germany). The light microscopy images were visualized in a microscope (Leica DM/LS, Leica Microsystems Ltd, Germany) connected to the image digitalization system (Leica Imaging Solutions, Leica Microsystems Ltd, Germany) and the digitalized images of the hearts were analyzed using software (Leica QWin Plus V.3.2.0., Leica Microsystems Imaging Solutions Ltd., Cambridge, UK). To estimate myocyte hypertrophy, the average nuclear volume of cardiomyocytes was measured in histological sections stained with hematoxylin and eosin, in areas remote from the infarction zone (septum and posterior wall) as previously published [10, 50–52]. For each animal studied, 50 nuclei were measured, evaluated in a total of 10–15 fields of 75,000 μm each, calculating the volume of each nucleus according to the equation [52]:

$${\text{Nuclear volume nuclear}}\left( V \right) \, = \, \pi \, x \, D \, x \, d^{2} / \, 6,$$

where *d* was the shorter diameter and *D* the longer diameter measured.

To evaluate the collagen content in the interstitium of the myocardium remote to the infarction, sections were mounted on slides and stained using the picrosirius red according to previously published [42]. The calibration of the optical and digital system of the Image Tool 3.0 software will allow us to analyze the images with an Olympus lens. Each image was captured with an UPlanFI 40 × objective. Ten to fifteen fields captured from the interventricular septum and posterior wall remote from the infarct scar of each animal were evaluated. In the case of the posterior wall, to ensure that the area analyzed was tissue remote from the infarction zone, at least five fields of vision moving away from the area occupied by the scar were disregarded, to then be considered an area of analysis. The results were expressed in percentage of the area occupied by collagen fibers in relation to the respective field of approximately 300,000 μm^2^ of area [51].

### Statistical analysis

Data are presented as means ± standard error of the mean. The normality of data distribution was assessed using the Shapiro–Wilk’s test. Bivariate comparisons were performed using Student's t test, for independent samples, and multivariate comparisons using 1-way or 2-way analysis of variance (ANOVA), complemented by Tukey's post hoc test if necessary. All analyzes were performed using Prism 8.0 (GraphPad Software Inc., San Diego, CA, USA) and SPSS for Windows (version 12.0, SPSS Inc., Chicago, Illinois, USA) programs, with a significance level set at *p* < 0.05.

## Results

### Effects of the strength training program on functional performance

**Fig. 1:**
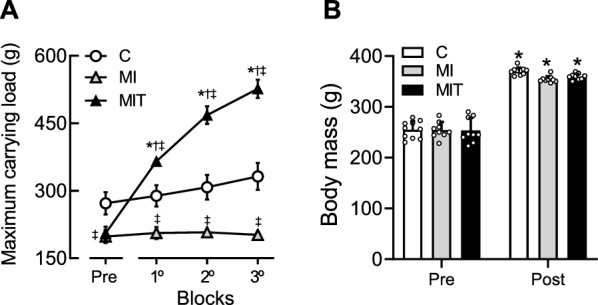
**A** Maximum carrying load measured before (Pre) and after the progressive blocks of the ST program of the infarcted and trained group (MIT, *n* = 10) compared with the untrained controls (C, *n* = 7) and infarcted (MI, *n* = 10) animals. **B** Body mass of groups measured before (Pre) and after training program (Post). Two-way ANOVA plus Tukey’s post hoc test in **A** (*p < 0.05 vs. Pre, ^†^p < 0.05 vs. MI, ‡p < 0.05 vs. C and **B** (*p < 0.05 vs. Pre). Symbols and bars represent mean ± SEM

The animals in the MIT group showed a progressive increase in the total load volume lifted in each block (1st: 32,514 ± 5,388 g, 2nd: 45,608 ± 3,237 g and 3rd: 55,250 ± 7,370 g; F_(1.320, 11.88)_ = 41.20; *p* < 0.001), with an average total load volume of 44,424 ± 10,900 g. Figure 1 shows the maximum mass-bearing capacity of all groups over the follow-up period: while the C and MI groups did not differ significantly from each other over the training protocol, the MIT animals exhibited a mean increase of 60 ± 3% in relation to the evaluation prior to the beginning of the protocol. The assessment of strength capacity at the beginning of the experimental program (that is, after the MI healing period) indicated that the animals from the MI and MIT groups had a lower maximum capacity to carry load than the C group (F = 4.827; *p* = 0.0173). Furthermore, the MI and MIT groups did not differ before the beginning of the physical training protocols, showing homogeneity in the randomization process. Furthermore, while the untrained groups (i.e., C and MI) did not have significant changes in the maximum load carried (C: F_(3, 24)_ = 0.9254; *p* = 0.4436 and MI: F_( 3, 36)_ = 0.1254; *p* = 0.9444), the MIT group showed a significant and progressive increase, which confirms the effectiveness in improving muscle strength over time (F_(3, 36)_ = 63.67; *p* < 0.0001).

Regarding the body mass, although an effect was found over time (F_(1, 25)_ = 635; *p* < 0.0001) indicating weight gain at the end of follow-up, as shown in Fig. 1B, it was not identified an effect of group (F_(2, 25)_ = 2.092; *p* = 0.1445). It is worth mentioning here that there were no animal deaths during follow-up.

### Effect of strength training program on the cardiac mass and lung water content

In Table 2, absolute and relative masses (indexed by respective body mass) of the atria increased in the infarcted groups compared to the C group. However, the increases in the cardiac mass observed after infarction were significantly mitigated by the ST program, where the absolute and relative masses of the right ventricles (RV) and left ventricles (LV) of the MIT group were smaller than those from the MI group. The lung water content, as an indicator of pulmonary congestion, significantly increased in the MI and MIT group in comparison to the C group. Nevertheless, the ST program significantly attenuated this index.

**Table 2:** Cardiac masses and lung water content

	C	MI	MIT	F	*p*
Atrial mass					
Absolute (mg)	27.6 ± 1.2	90.8 ± 13.6*	82.2 ± 15.6*	9.06	0.0011
Relative (mg/g)	0.07 ± 0.01	0.25 ± 0.03*	0.23 ± 0.04*	9.30	0.0010
RV mass					
Absolute (mg)	156.2 ± 8.2	296.1 ± 29.9*	222.2 ± 18.7^†^	12.10	0.0002
Relative (mg/g)	0.42 ± 0.02	0.83 ± 0.08*	0.61 ± 0.05^†^	12.46	0.0002
LV mass					
Absolute (mg)	487.6 ± 9.4	793.1 ± 19.3*	673.0 ± 6.1*^†^	150.1	< 0.0001
Relative (mg/g)	1.31 ± 0.02	2.24 ± 0.05*	1.87 ± 0.02*^†^	170.2	< 0.0001
Lung water (%)	77.80 ± 0.46	81.67 ± 0.50*	79.89 ± 0.26*^†^	98,22	< 0.0001

Values are means ± SEM of control (C, *n* = 10), infarcted (MI, *n* = 9) and infarcted and trained (MIT, *n* = 9) groups. Absolute masses of the cardiac chambers were indexed by the respective body mass (relative, mg/g of body mass)

RV, right ventricle. LV, left ventricle

*p < 0.05 vs. C. ^†^*p* < 0.05 vs. MI, by 1-way ANOVA plus Tukey’s post hoc

### Morphofunctional analysis by Doppler echocardiography

No differences were found in infarct size between the MI (45.2 ± 1.5% of LV) and MIT groups (45.4 ± 1.8% of LV) after 12 weeks of ST program (t = 0.082; 95% CI − 4.879–5.279; *p* = 0.9350). However, significant changes were observed in the cardiac structural and functional parameters (Table 3). Although the LV areas were larger in both infarcted groups compared to the C group, the MIT group exhibited diastolic and systolic areas of the LV significantly smaller than MI animals. Similarly, the ST program also partially preserved the functional parameters impacted by the MI. The LV fractional shortening was higher in the MIT groups compared to MI, even when both groups had reduced values compared to the C group. The A and E wave velocities were changed in the MI compared to C animals, but not significantly in the MIT groups. In addition, the elevation of the E/A ratio observed in the MI group was not present in infarcted-trained rats.

**Table 3:** Echocardiographic parameters of left ventricle after strength training program

Parameters	C	MI	MIT	F	*p*
Structural					
LVDA (mm)	26.3 ± 1.8	47.3 ± 5.8*	37.5 ± 2.1*^†^	80.90	< 0.0001
LVSA (mm)	12.2 ± 0.7	37.3 ± 5.6*	26.3 ± 2.2*^†^	126.2	< 0.0001
Functional					
E (m/s)	73.6 ± 9.8	89.9 ± 13.3*	80.5 ± 12.8	4.58	0.0193
A (m/s)	28.2 ± 5.6	18.4 ± 2.4*	27.5 ± 5.8^†^	12.58	0.0001
E/A	2.71 ± 0.71	5.18 ± 0.54*	2.80 ± 0.42^†^	59.85	< 0.0001
LVFS (%)	53.4 ± 3.1	21.4 ± 3.9*	29.8 ± 7.3*^†^	104.7	< 0.0001

Values expressed as mean ± SEM of control (C, *n* = 10), infarcted (MI, *n* = 10) and infarcted and trained (MIT, *n* = 10) groups

LVDA: left ventricle diastolic area, LVSA: left ventricle systolic area, E: E wave velocity, A: A wave velocity, E/A: E to A ratio, LVFS: LV fractional shortening

*p < 0.05 vs. C. ^†^*p* < 0.05 vs. MI, by 1-way ANOVA plus Tukey’s post hoc

### Effects of the strength training program on left ventricular hemodynamics

Table 4 shows the LV hemodynamics data from the groups assessed at the end of follow-up. No differences were observed between HR values between groups. By direct catheterization of the LV, a reduction of the systolic intraventricular pressure was observed in the MI group that was totally prevented by the ST program. LV end-diastolic pressure (LVEDP) was elevated in both infarcted groups, although MIT had LVEDP significantly lower than untrained MI rats. Similar behavior was found in the first derivative of pressure (dP/dt_max_): +dP/dt_max_ and -dP/dt_max_ and were impaired by infarction, but the ST program partially preserved these parameters of global cardiac function. Finally, no differences were observed in HR values between groups.

**Table 4:** Hemodynamics of left ventricle and myocardial mechanics after strength training program

Parameters	C	MI	MIT	F	*p*
Hemodynamics					
HR (bpm)	357 ± 33	344 ± 26	333 ± 21	1.76	0.1914
LVSP (mmHg)	117 ± 7	105 ± 7*	113 ± 6^†^	8.47	0.0015
LVEDP (mmHg)	3 ± 1	17 ± 7*	10 ± 5*^†^	17.62	< 0.0001
+dP/dt_max_ (mmHg/s)	8,863 ± 763	4,716 ± 1,211*	7,355 ± 1,408*^†^	33.35	< 0.0001
-dP/dt_max_ (mmHg/s)	6,090 ± 551	3,602 ± 715*	4,959 ± 754*^†^	33.97	< 0.0001
Myocardial mechanics					
DT (g/mm^2^)	6.24 ± 0.27	2.36 ± 0.28*	3.81 ± 0.24*^†^	54.12	< 0.0001
RT (g/mm^2^)	1.04 ± 0.09	1.00 ± 0.06	1.07 ± 0.08	0.13	0.8716
+dT/dt_max_ (g/mm^2^/s)	61.56 ± 4.75	18.83 ± 2.85*	35.01 ± 1.64*^†^	39.46	< 0.0001
-dT/dt_max_ (g/mm^2^/s)	45.47 ± 3.22	11.32 ± 1.66*	21.94 ± 1.33*^†^	57.97	< 0.0001
TPT (ms)	174.00 ± 7.02	259.90 ± 9.74*	233.30 ± 10.37*	23.89	< 0.0001
TR_50%_ (ms)	139.77 ± 7.72	217.20 ± 14.36*	204.40 ± 12.84*	13.02	0.0001

Values expressed as mean ± SEM of the control (C, *n* = 10), infarcted (MI, *n* = 10) and infarcted and trained (MIT, *n* = 9) groups.

HR: heart rate, LVSP: left ventricular systolic pressure, LVEDP: left ventricular end-diastolic pressure, +dP/dt_max_: maximum rate of positive variation in LV pressure, -dP/dt_max_: maximum rate of negative variation in developed pressure, DT: developed tension, RT: rest tension, +dT/dt: maximum rate of positive variation of tension, -dT/dt_max_: maximum rate of negative variation of tension, TPT: time to peak of tension. TR_50%_: time for RT to decay by 50%

*p < 0.05 vs. C. ^†^*p* < 0.05 vs. MI, by 1-way ANOVA plus Tukey’s post hoc

### Effects of the strength training program on “in vitro” myocardial contractility

There were no significant differences in the lengths (C: 7.37 ± 1.00 mm, MI: 5.40 ± 0.71 mm, MIT: 5.38 ± 0.58 mm; F = 2.77, *p* = 0.0814) and masses (C: 5.40 ± 0.71 mg, MI: 6.34 ± 0.63 mg, MIT: 5.17 ± 0.45 mg; F = 0.97, *p* = 0.3912) of the papillary muscles isolated from the experimental groups. However the muscle cross-sectional area was larger in the MI group than the C group, while MIT had intermediary values (C: 0.73 ± 0.05 mm^2^, MI: 1.21 ± 0.07 mm^2^, MIT: 0.98 ± 0.03 mm^2^, F = 18.94, *p* < 0.001).

Myocardial mechanics evaluated at steady-state condition are shown in Table 4. Cardiac muscles from MI rats exhibited depressed maximal developed tension (DT) and its time-derivative when compared with the C group, while MIT muscle mechanics were partially preserved with parameters significantly better than MI, although it was still lower than the C group. For the time parameters of contraction and relaxation (i.e., TPT and TR_50%_), the ST program was not able to interfere in the changes caused by infarction, which means that values of the MI and MIT groups were similar and both groups had longer times to contract and relax when compared to C. No differences were observed in RT values between groups.

**Fig. 2:**
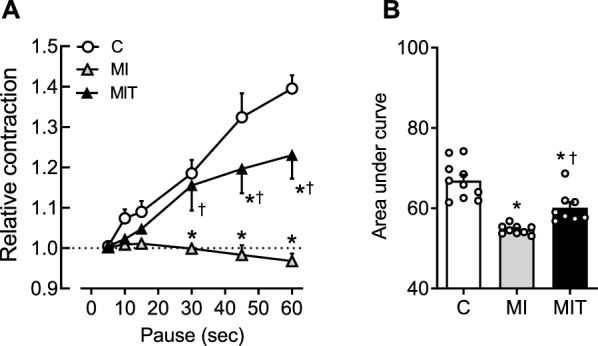
Panel **A**: effect of increasing pause durations on the relative contraction (post-rest contraction divided by previous twitch of tension) of the control (C, *n* = 10), infarcted (MI, *n* = 9) and infarcted and trained (MIT, *n* = 8) groups. Panel **B**: area under curves calculated from the sided graphic. In **A**, *p < 0.05 vs. C. ^†^*p* < 0.05 vs. MI, by 2-way ANOVA plus Tukey’s post hoc. In **B**, *p < 0.05 vs. C. ^†^*p* < 0.05 vs. MI, by 1-way ANOVA plus Tukey’s post hoc. Symbols and bars represent mean ± SEM

Under the use of different protocols for analysis of myocardial performance, additional significant effects of the ST program were noted. As shown in Fig. 2, pauses in the electrical stimulus induced potentiation of developed tension when stimuli were restarted, being this effect proportional to the pause duration. However, compared to the C group, this behavior was blunted in the muscles isolated from the MI group, having even a decay of force after each pause in relation to the steady-state contractions. On the other hand, the MIT group showed partially preservation of the post-pause potentiation of force. To simplify the analysis, the area under the curve (AUC) was calculated for each experiment and as shown in Fig. 2B, there was a reduction on the AUC of the group MI compared to C, while MIT exhibited AUC smaller than C but significantly larger than MI (F_(2, 25)_ = 14.92; *p* < 0.0001).

**Fig. 3:**
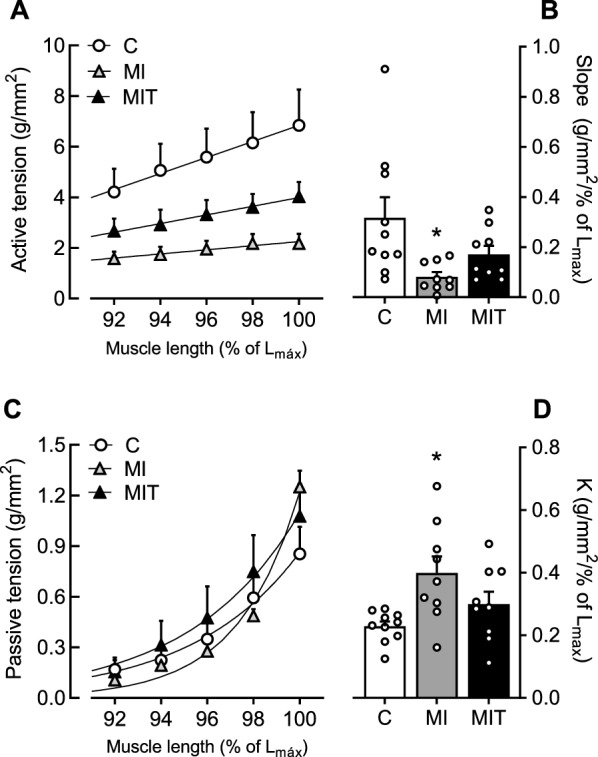
Data of active tension (**A**–**B**) and passive tension (**C**–**D**) in response to muscle stretching of the control (**C**, *n* = 10), infarcted (MI, *n* = 9) and infarcted and trained (MIT, *n* = 9) groups. Bars represent slopes of the linear regressions (**B**) or growth rate constant of the exponential function (**D**). In **B** and **D**, *p < 0.05 vs. **C**, by 1-way ANOVA plus Tukey’s post hoc. Symbols and bars represent mean ± SEM

In response to stretching (Fig. 3A), the best fit to the length–tension relationship resulted in a linear regression indicating that increase in the active tension was found for each stretch. As shown in Fig. 3B, significant differences were observed only between groups (F(2, 25) = 4.841; *p* = 0.0167): the cardiac muscle isolated from the MI rats exhibited flattened responses with lower slopes (0.08 ± 0.01 g/mm^2^/%L_max_) than those from the C group (0.31 ± 0.08 g/mm^2^/%L_max_). However, this linear coefficient of the MIT group (0.16 ± 0.03 g/mm^2^/%L_max_) was intermediary and not statistically different from either C or MI slopes. The passive tension also increased as a function of muscle stretching, but the relationship between stretching and passive tension had exponential function as better fitting (Fig. 3C). As a result, Fig. 3D shows differences were evaluated as a function of the growth rate constant (K) that was higher in the MI group than C (F_(2, 25)_ = 4.785; *p* = 0.0174; C: 0.22 ± 0.01 g/mm^2^/%L_max_; MI: 0.39 ± 0.05 g/mm^2^/%L_max_) suggesting an increased stiffness, while the MIT was partially preserved (0.29 ± 0.03 g/mm^2^/%L_max_).

**Fig. 4:**
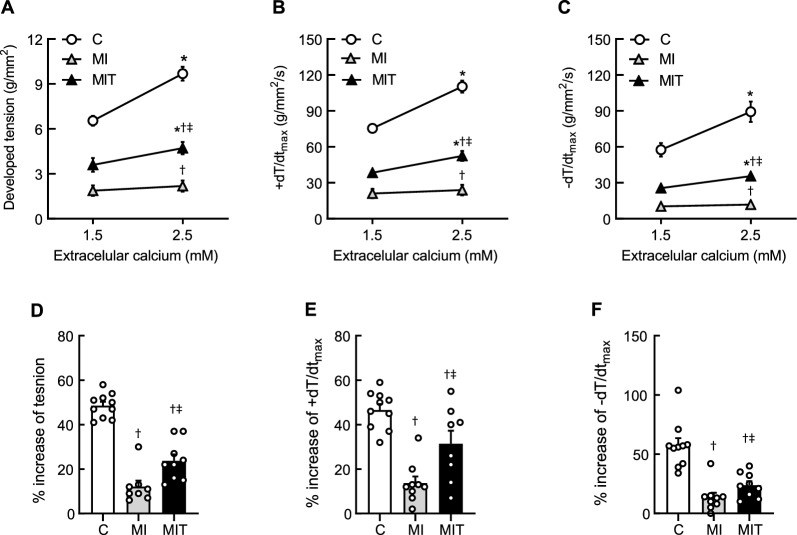
Effect of increasing the concentration of calcium in the nutrient solution on the contractile parameters of the isolated papillary muscle in the control (**C**, *n* = 10), infarcted (MI, *n* = 9) and infarcted and trained (MIT, *n* = 9) groups. In A-**C**, the effects on developed tension, maximal positive (+ dT/dt_max_) and negative (-dT/dt_max_) time-derivatives of tension are represented. In **D**–**F**, the percent of increase in respective contractile parameters as result of the increasing calcium concentration. Two-way ANOVA plus Tukey’s post hoc test in **A**–**C** (*p < 0.05 vs. Ca^2+^ 1.5 mM, ^†^*p* < 0.05 vs. **C**, ‡*p* < 0.05 vs. MI) and 1-way ANOVA plus Tukey’s post hoc test in **D**–**F** (^†^*p* < 0.05 vs. **C**, ‡*p* < 0.05 vs. MI). Symbols and bars represent mean ± SEM

The increase in the Ca^2+^ concentration in the nourishing solution from 1.5 mM to 2.5 mM stimulated the contractility of papillary muscle from all groups, as shown in Fig. 4. The increase in developed tension in response to Ca^2+^ was insignificant in the MI group in relation to the response in the C group, while in the MIT group this response was partially preserved. Significant differences were found after changing the Ca^2+^ from 1.5 mM to 2.5 mM in C (before Ca^2+^ change: 6.54 ± 0.33 g/mm^2^ vs. after: 9.68 ± 0.46 g/mm^2^; MD[95% CI] − 3.14[− 3.45–− 2.84]; *p* < 0.05) and MIT groups (before Ca^2+^ change: 3.59 ± 0.47 g/mm^2^ vs. after: 4.37 ± 0.54 g/mm^2^; MD[95% CI] − 0.78[− 1.10–− 0.46]; *p* < 0.05), but not in the MI group (before Ca^2+^ change: 1.87 ± 0.25 g/mm^2^ vs. after: 2.18 ± 0.30 g/mm^2^; MD[95% IC]: − 0.30[− 0.62–0.01]; *p* > 0,05). Similarly, the positive derivative of tension increased in response to change of calcium concentration in the C group (before Ca^2+^ change: 75.4 ± 3.4 g/mm^2^; after: 110.3 ± 4.9 g/mm^2^; MD[95% IC]: − 34.93[− 41.73–− 28.13]; *p* < 0.05) and actually did not significantly change in the MI group (before: 21.0 ± 3.9 g/mm^2^, after: 23.9 ± 4.4 g/mm^2^; MD[95% CI]: − 2.87[− 10.04–4.29]; *p* > 0.05). However, it was partially preserved in the MIT group (before: 38.2 ± 2.6 g/mm^2^; after: 48.3 ± 4.6 g/mm^2^; MD[95% IC]: − 7.86[− 15.03–− 0.69]; *p* < 0.05). Finally, the analysis of -dT/dt_max_ in response to elevation on the Ca^2+^ concentration showed significant lusitropic response only in C (before Ca^2+^ change: 57.6 ± 5.7 g/mm^2^, after: 89.2 ± 8.6 g/mm^2^; MD[95% CI]: − 31.63[− 37.29–− 25.98]; *p* < 0.05) and MIT group (before: 27.4 ± 2.1 g/mm^2^, after: 33.6 ± 2.1 g/mm^2^; MD[95% CI]: − 6.18[− 12.15–− 0.22]; *p* < 0.05), without significant increase on the MI group (before: 10.3 ± 1.8 g/mm^2^; after: 11.8 ± 2.1 g/mm^2^; MD[95% CI]: − 1.48[− 7.44–4.47]; *p* > 0.05).

### Effects of the strength training program on histomorphometry of the myocardium remote to infarction

**Fig. 5:**
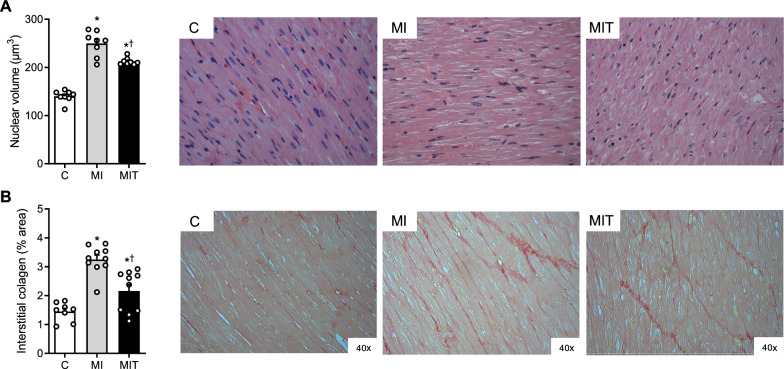
Representative microphotographs of the remote remodeled myocardium stained with eosin–hematoxylin to nuclear volume mensuration (upper pictures) and eosin–picrosirius red for collagen occupied area evaluation (lower pictures). Remodeling parameters included myocyte hypertrophy as estimated by averaged nuclear volume (**A**) and interstitial fibrosis estimated by % of area occupied by collagen fibers (**B**) evaluated in the control (**C**, *n* = 8), infarcted (**MI**, *n* = 10) and infarcted and trained (**MIT**, *n* = 10) groups. *p < 0.05 vs. **C**. ^†^*p* < 0.05 vs. **MI**, by 1-way ANOVA plus Tukey’s post hoc. Symbols and bars represent mean ± SEM

Figure 5 represents the nuclear volume of the cardiomyocytes measure in the reminiscent myocardium, used as an index of hypertrophy. There were significant increase in both infarcted groups when compared to the nuclei volume assessed in equivalent regions of myocardium tissue from the C group (Fig. 5A). However, the MIT group presented statistically smaller nuclear volumes than the untrained MI group, suggesting a partial prevention of the hypertrophic remodeling. Similarly, the percent of collagen fibers occupying the myocardium remote to the infarct area (Fig. 5B) was greater in the MI group, while interstitial fibrosis of the remodeled tissue was partially prevented in the MIT group.

## Discussion

### Methodological aspects

Although the techniques used in the present study are well recognized, some methodological issues are worth noting. Despite MI studies in animal models are consistently performed in different species including rats [53, 54], mice [55, 56], rabbits [56] and pigs [57], we chose to use rats due to the higher probability of developing HF after the acute ischemic insult. Based on the technique originally developed by Heimburger [58], the procedure has been improved over the decades and is widely used currently [42, 53, 59, 60]. Another issue is the choice of lineage and sex of the animals. Most work with rats uses Wistar or Sprague–Dawley strains. For this study, Wistar rats were used, and are currently the most used in animal facilities, and frequently used in experimental research for MI induction [61–63]. Although inconclusive, the post-MI pathophysiological process points to important distinctions between the sexes of the animals [64] due to estrogen-related cardioprotection, allowing mild cardiac dysfunction and remodeling in females if compared to males, as well as the upregulated integrin-linked kinases that provide some myocardial regeneration [65], increased angiogenesis and reduced myocyte apoptosis [66] in females. Thus, in the present study we chose to use male rats to avoid the cardioprotective effects in females acting as a confounding factor and promoting inconsistent findings.

Moreover, the method to produce MI is also worth mentioning. Although other models are available in the related literature [67, 68], the permanent surgical occlusion of the anterior intraventricular branch of the left coronary artery is the most used strategy in experimental research [53]. By using this technique, myocardial necrosis that follows induces rapid hemodynamic overload and cardiac decompensation that reproduce complications that mimic those found in humans, allowing evaluations on the processes enrolled in the ischemic event, cardiac remodeling, and HF development, as well as potential therapeutic opportunities [53].

However, despite the advantages of the method, surgical occlusion of the coronary artery can lead to different infarct sizes and, consequently, different cardiac remodeling and ventricular dysfunction. In fact, Tucci [53] reports that infarcts greater than 40% of the LV are associated with relevant cardiac morphofunctional repercussions, such as severe cardiac dysfunction with higher end-diastolic volume/pressures, decreased cardiac output and significant pulmonary congestion. On the other hand, smaller infarcts cause only mild changes on the ventricular function and atrial emptying and, therefore, do not lead to pulmonary congestion or any repercussions to the RV. Additionally, dos Santos [42] reported that rats with MI smaller than 35% of the LV often could present hemodynamic parameters normal or very similar to normal, complicating the appropriate characterization of cardiac dysfunction. Thus, as a strategy to minimize the possible effects that different MI sizes could produce, only those animals with MI larger than 35% of the LV comprised the infarcted groups. In the present study, no significant differences were observed in the infarct size of the MIT and MI groups before the initiation of ST program (47,1 ± 5,6 vs. 45,0 ± 3,9% of the LV, data not shown), which demonstrates homogeneity and adequate randomization. Furthermore, at the end of protocols, the infarct size of these groups remained similar, indicating that differences observed were not a result of exercise changes on the infarct size.

### Effects of strength training

Several ST protocols are available for rodents, including platform jumping [69, 70], squatting [71, 72] and climbing [62, 73, 74]. In this study, we opted for climbing due to its effectiveness in promoting important neuromuscular adaptations such as skeletal muscle hypertrophy and increased physical capacity [45, 75, 76] without causing serious injuries or death (Krause [77]. Lourenço et al. [78] performed a meta-analysis of studies that used climbing and reinforced that, in fact, such a procedure is effective in increasing muscle mass, as well as maximum strength. In these ST protocols, factors such as the number of repetitions (depending on the height of the stairs and distance between the steps), the total number of climbs in the session (depending on the number of series and duration of intervals), the weekly frequency of training, as well as the duration of the program. However, even if these variables are considered simple, it is possible to recognize numerous possibilities in the organization of the training program.

Considering ST programs in infarcted animals, there are few data in the literature. Grans et al. [19] that used 12 weeks of training, 5 days a week with an intensity of 40–60% of the maximum load capacity. Meanwhile, more recently Garza et al. [73] applied a 10-week training program, with an initial intensity of 50% of the maximum load that was intensified by 5% at each climb totaling 8–10 climbs per session, with 2-min break between sets. In the present study, the animals were submitted to a training program with greater volume (given by weekly frequency and number of climbs) and longer duration, but with a block-based organization. Fundamentally, this design was based on previous findings by our group [46, 79], which demonstrated that spontaneously hypertensive animals submitted to the same ST program had attenuation of arterial hypertension and preservation of ventricular function, without major changes in cardiac mass.

Regarding the improvement in skeletal muscle endurance, there was a 60% increase in the maximum load capacity of infarcted and trained rats, greater to that reported by Grans et al. [19] in infarcted rats, and similar to others using hypertensive rats [46] or a postmenopausal rat model [79]. A point that needs consideration corresponds to the effect of this design on the presence or not of any previous involvement. As previously mentioned, there are few studies that have analyzed the influence of strength training in infarcted rats [19, 73], although only Grans et al. [19] reported some results on increased skeletal muscle strength. Therefore, our study reinforces current knowledge and clinical data in the literature that indicates a relevant role for this type and training in improving capacity, especially in CVD.

In this perspective, Perilhão et al. [46] demonstrated that there is a correlation between strength gain and blood pressure reduction in spontaneously hypertensive rats, indicating that gains in strength may be correlated with better clinical conditions in CVD. Clinically, this association between improved strength and health conditions is already well established, including evidence of reduced morbidity and mortality in HF patients [80].

Notwithstanding, a minimal parcel of the current literature on the effects of ST in animal models of CVD is concerned with calculating total training volume. Actually, despite numerous proposals to calculate the volume, few studies have addressed this parameter [46, 74]. In the present study the training volume could be calculated by multiplying the load used, the number of repetitions and the series number, as previously proposed [46, 74, 81]. This is relevant, as Krause Neto et al. [81] demonstrated that the adaptations promoted by ST on the development of strength and muscle hypertrophy in elderly rats could be explained by the total load of the protocol. Indeed, as training volume plays a key role in strength training adaptations, it should be imperative that this approach be considered for studies in this field. For example, Krause Neto et al. [74] suggested that ST protocols with the aim of promoting muscle hypertrophy need to be based on volume scaling, maintaining a positive correlation between muscle hypertrophy and load progression, training volume and total load, since a program based on climbing to exhaustion may limit morphological adjustments.

### Effects of strength training program on body mass

At the beginning of the protocol, all animals were weighed before the surgical procedures for coronary occlusion or sham surgery, with no differences between groups were identified. Similarly, 12 weeks after the randomization to participate in the ST protocol or stay at rest, there were also no differences between the body mass of the experimental groups. Our results are similar to the findings of other studies [82, 83], however, unlike others that demonstrated that infarcted animals had greater body mass [62], whose differences could be explained by the age diversity of the rats used, as well as control of caloric intake and follow-up time. In the present study, the animals are considered young adults [84] and the caloric intake of the animals was not controlled.

### Effects of the strength training program on global cardiac function and pulmonary congestion

In the present study, the MI animals exhibited reduced fractional shortening and elevated E/A ratio by echocardiography, associated with increased pulmonary water content compared to the C group, which is in agreement with the large infarct size. In fact, the percentage of pulmonary water content, as an estimate of congestion, is a strong indicator of HF [53, 82, 85–89]. On the other hand, there was an attenuation of pulmonary congestion in the MIT group, a fact that has already been identified by other studies involving different models of physical training in infarcted rats [62, 82]. In the clinical scenario, these findings are relevant and reinforce the protective role of exercise, especially due to the evidence that indicates that greater pulmonary congestion is associated with worse prognosis and high mortality rate [90].

There are only two previous studies available that tested the effects of ST by climbing in infarcted animals [19, 73]. Grans et al. [19] submitted infarcted rats to 12 weeks of training with 15 daily series under 40–60% of the maximum load carried, 5 days a week, and as in our study, found an increase in strength associated with a reduction in the E/A ratio and cardiac performance by echocardiography, in addition to additional benefits on cardiovascular autonomic control. Likewise, despite using different structure of the ST program (10 weeks of training with progressive increase in intensity at each session of 8–10 climbs), Garza et al. [73] also showed partial preservation of the shortening by echocardiography and improvement in systolic and diastolic hemodynamic performance assessed by positive and negative derivatives of ventricular pressure. Thus, our echocardiographic and hemodynamic data corroborate these previous findings on the ladder-based ST-related benefits regarding cardiac dilatation and dysfunction. However, this remarkable effect should not be credited to a reduction in infarct size, but rather to actions in remodeling the tissue reminiscent of the infarct.

### Effects of the strength training program on cardiac adverse remodeling

The histological data present in this study may be considered an important point of theme, since the it topic is not fully clarified on literature. As expected, the cardiac mass of the infarcted animals was greater than that of the control group, which was confirmed by histology, showing cardiomyocyte hypertrophy evaluated in the myocardium remote from the MI area. From this perspective, it is worth mentioning that post-MI remodeling occurs asymmetrically, with hypertrophy of the remaining muscle, an important contributor to ventricular dysfunction [40, 91]. It is known that after MI, despite the loss of contractile tissue in the ischemic area, there is an overload and consequent hypertrophic remodeling of the reminiscent issue, associated with an intense inflammatory process and collagen accumulation, leading the total cardiac mass to increase. According to our data, the LV mass indexed by body mass was 60% greater in the MI group when compared to the C group, similar to the information already published by our laboratory and by other authors, in animals with infarct sizes greater than 35% of the LV after 6 weeks of surgery [14, 73, 92–97]. Cardiomyocyte hypertrophy is described by the increase in cell and nucleus size, and the production and reorganization of its components [52]. In this sense, the increase in the nuclear volume of cardiomyocytes in the infarcted animals in the present study confirms that myocardial hypertrophy was indeed present.

Regarding the effects of the ST program on this parameter, our data revealed an attenuation in the post-MI increase in cardiac chamber mass and also in the cardiomyocyte hypertrophy, which is in agreement with the other functional results obtained, since ventricular hypertrophy is associated with ventricular dysfunction and higher morbidity and mortality both in humans [98] and in animal models. This effect of the hypertrophic process present in post-MI adverse remodeling has already been reported in studies with rats that performed aerobic exercise by swimming [82] or running [19, 99]. To our knowledge, only one previous study [19] suggested attenuation in the cardiac mass estimated by echocardiography in infarcted animals after ST, and the present study is the first to demonstrate a protective effect of a ST program on post-MI hypertrophy by weighing the cardiac chambers and performing histological analysis of the cardiac tissue. Although the present study did not aim to evaluate molecular pathways involved in this ST effect, it was previously proposed that physical training is capable of downregulating essential proteins in pathways involved in pathological hypertrophy [17].

As mentioned, the remodeling of the tissue remaining after the infarction is also accompanied by excessive synthesis and deposition of interstitial collagen fibers. Eighteen weeks after the infarction, we evidenced interstitial fibrosis estimated by the increase in the degree of collagen deposition in both infarcted groups, which agrees with previous studies [97]. In fact, cardiac dysfunction that follows post-MI remodeling depends both on changes in the cardiomyocyte and those that also occur in the extracellular matrix, mainly with regard to myocardial stiffness and diastolic dysfunction. Our results indicate that the ST program partially prevented this increase in collagen in the myocardial tissue, and the present study is unique in demonstrating this effect resulting from this type of physical training. Despite this information gap about the mechanisms involved, it is possible to speculate that similarly to that proposed by a study that used endurance training in infarcted rats [100, 101], a reduction in the activation of myofibroblasts and in metalloproteinases may play a key role in attenuating myocardial collagen deposition. Thus, as the uncontrolled accumulation of the extracellular matrix by little elastic structural proteins such as type I collagen can substantially compromise myocardial compliance, this could be the structural substrate of the reason why the ST program was able to alleviate diastolic dysfunction and the exacerbated passive tension response to stretching of the papillary muscle of the infarcted animals.

### Effects of the strength training program on myocardial contractile function

Myocardial mechanics were evaluated considering the important role of remnant tissue in overall cardiac performance. The "in vitro" study of myocardial contractility can be performed using several methods, with their respective advantages and disadvantages. The analysis of papillary muscles to evaluate myocardial contractility in rats has been used in several studies [3, 102–106]. In studies involving infarction, multicellular preparations of the myocardium, such as that of the isolated papillary muscle remote from the scar, exempt the interpretations obtained from possible influences of ventricular geometry and the infarcted tissue itself, maintaining exclusively the mechanical properties of the remaining tissue [47, 48, 51]. In this situation, adverse functional remodeling is characterized by reduced contractile capacity [107] in addition to depression of the inotropic and lusitropic responses and prolongation of temporal parameters [3, 92, 108, 109]. The level of contractile dysfunction found in our study was similar to that from other studies [11–13, 63, 92, 93, 108–111] and equivalent to identified in isolated cardiomyocytes [13, 63].

Considering the effects of the ST program, our data on myocardial contractility parameters in infarcted rats are unprecedented. Although ST is considered an effective adjuvant approach in preserving muscle mass, improving strength and endurance, as well as increasing maximum oxygen consumption, which justifies the improvement in quality of life in post-infarction patients [18, 19, 23–25], there were no previous studies on the effects of this type of training on the functional myocardial mechanisms. In summary, our findings indicate that ST partially preserved myocardial contractility, associated with positive effects on the contractile response to pause and to Ca^2+^ when compared to the cardiac muscle isolated from the untrained MI rats.

Among the mechanisms that explain the contractile dysfunction of the post-MI remodeling, the most studied are those changes in the expression of many proteins involved in the Ca^2+^ cycle and in the myofibrils [53, 54, 112]. Impairments in the contraction and relaxation of cardiomyocytes isolated from areas remote from the infarction have been described [11, 11, 12, 12, 13, 13, 63], including decreases in systolic calcium and elevation of diastolic Ca^2+^ in the myoplasm [17], that occur in parallel with the decrease in the trans-sarcolemmal Ca^2+^ influx current through the L-type channels [104, 113–115] and an impairment in Ca^2+^ reuptake by the sarcoplasmic reticulum (SR) by Ca^2+^-ATPase (SERCA2) [63].

Although the expression of proteins involved in the management of Ca^2+^ in the cardiomyocyte has not been evaluated, in the present study the "in vitro" protocol was used to analyze the potentiation of post-rest strength, through which it is possible to indirectly evaluate the behavior (or disclose abnormalities) of Ca^2+^ kinetics on the excitation–contraction coupling [116]. This potentiation is due to the increase in Ca^2+^ uptake by the SR during the pause and consequent greater fractional release of Ca^2+^ during the post-pause contraction, which overlap the extrusion capacity of Ca^2+^ through the sarcolemma [117]. Thus, while this maneuver indirectly evaluates the Ca^2+^ cycle in the cardiac muscle [116–118], in contrast, myocardium samples from infarcted rats exhibit reduction of the post-pause contraction that is attributed to impaired Ca^2+^ uptake by the SR and excessive Ca^2+^ efflux through Na/Ca exchange. As expected, our results indicated impairment in the MI group in contrast to the clear potentiation of the contraction after increasing stimulus pauses in the C group. However, an important attenuation of this impairment was observed in the MIT group. Specifically, when evaluating the contraction after the longest pause (i.e., 60 s), the decay of force after pause in the MI group was converted back into potentiation by the ST program.

Additionally, the improvement in cardiac muscle contraction against high calcium concentrations, identified in the MIT group, can be interpreted in an integrated manner, indicating that both the kinetics and the responsiveness to calcium were partially preserved by the ST program. Together, these results could be the result of an improvement in the molecular remodeling of the proteins involved in the Ca^2+^ cycle, because of physical training.

In fact, supporting this hypothesis of the protective role of the ST on myocardial contractility, classic studies by Kemi et al. [119, 120] showed that an increase in SERCA expression can improve inotropism by increasing the accumulation of Ca^2+^ in the SR. Furthermore, studies by Zhang et al. [13, 63] indicated that physical training could accelerate contraction and improve calcium kinetics in isolated cardiomyocytes (increased reuptake and SR content) by modulating SERCA-2 expression of its deregulating peptide, phospholamban, and the Na/Ca exchanger. However, even if our functional findings are like those already reported in the literature using other training models, future studies should be carried out to assess the molecular mechanism underlying the protective effects of the ST program on the remodeled myocardium after MI, demonstrated in the present study.

## Conclusion

The infarcted animals submitted to a structured ST program with linear periodization showed an increase in the capacity to carry mass, associated with attenuation of cardiac remodeling (i.e., myocardial hypertrophy and intestinal fibrosis) and pulmonary congestion. The improvement in cardiac function can be attributed, at least in part, to the partial preservation of myocardial contractility assessed "in vitro" and the inotropic response to stimulus pauses and to the increase in calcium concentration.

### Supplementary Information

**Additional file 1**: File with raw data about body mass before MI sugery, and before and after ST protocol.

**Additional file 2**: File with raw data about active and passive lenght-tension curves of the isolated cardiac muscles.

## Data Availability

The datasets used in the present study are available from the corresponding author upon reasonable request.

## References

[1] Virani SS, Alonso A, Aparicio HJ, Benjamin EJ, Bittencourt MS, Callaway CW, Carson AP, Chamberlain AM, Cheng S, Delling FN, Elkind MSV, Evenson KR, Ferguson JF, Gupta DK, Khan SS, Kissela BM, Knutson KL, Lee CD, Lewis TT, Liu J, Loop MS, Lutsey PL, Ma J, Mackey J, Martin SS, Matchar DB, Mussolino ME, Navaneethan SD, Perak AM, Roth GA, Samad Z, Satou GM, Schroeder EB, Shah SH, Shay CM, Stokes A, VanWagner LB, Wang N-Y, Tsao CW (2021). Heart disease and stroke statistics—2021 update. Circulation.

[2] Long L, Mordi IR, Bridges C, Sagar VA, Davies EJ, Coats AJS, Dalal H, Rees K, Singh SJ, Taylor RS (2019). Exercise-based cardiac rehabilitation for adults with heart failure. Cochrane Database Syst Rev.

[3] Bocalini DS, Carvalho EVA, Mello AF, de Sousa R, Levy F, Tucci PJF (2010). Exercise training-induced enhancement in myocardial mechanics is lost after 2 weeks of detraining in rats. Eur J Appl Physiol.

[4] Jakovljevic B, Turnic TN, Jeremic N, Savic M, Jeremic J, Srejovic I, Belic B, Ponorac N, Jakovljevic V, Zivkovic V (2019). The impact of high-intensity interval training and moderate-intensity continuous training regimes on cardiodynamic parameters in isolated heart of normotensive and hypertensive rats. Can J Physiol Pharmacol.

[5] Negrão CE, Maria UP, Rondon B (2001). Exercício Físico, Hipertensão e Controle Barorreflexo Da Pressão Arterial. Revista Brasileira de Hipertensão.

[6] Polito MD, Farinatti PTV (2003). Respostas de Frequência Cardíaca, Pressão Arterial e Duplo-Produto Ao Exercício Contra-Resistência: Uma Revisão Da Literatura. Revista Portuguesa de Ciências Do Desporto.

[7] do Luiz PW, Lofrano MC, Oyama LM, Dâmaso AR (2009). Obesidade e adipocinas inflamatórias: implicações práticas para a prescrição de exercício. Revista Brasileira de Medicina Do Esporte.

[8] Silva F, de Jesus F, Drummond R, Fidelis MR, Freitas MO, Leal TF, Teixeira LM, de Rezende A, de Moura G, Reis ECC, Natali AJ (2021). Continuous aerobic exercise prevents detrimental remodeling and right heart myocyte contraction and calcium cycling dysfunction in pulmonary artery hypertension. J Cardiovasc Pharmacol.

[9] Teodoro BG, Natali AJ, do FernandesPeluzio SATMCG (2010). A influência da intensidade do exercício físico aeróbio no processo aterosclerótico. Revista Brasileira de Medicina Do Esporte.

[10] Serra AJ, Santos MHH, Bocalini DS, Antônio EL, Levy RF, Santos AA, Higuchi ML, Silva Jr JA, Magalhães FC, Baraúna VG, Krieger JE, Tucci PJF (2010). Exercise training inhibits inflammatory cytokines and more than prevents myocardial dysfunction in rats with sustained β-adrenergic hyperactivity. J Physiol.

[11] Zhang L-Q, Zhang X-Q, Musch TI, Moore RL, Cheung JY (2000). Sprint training restores normal contractility in postinfarction rat myocytes. J Appl Physiol.

[12] Zhang L-Q, Zhang X-Q, Ng Y-C, Rothblum LI, Musch TI, Moore RL, Cheung JY (2000). Sprint training normalizes Ca (2+) transients and SR function in postinfarction rat myocytes. J Appl Physiol.

[13] Zhang LQ, Zhang XQ, Ng YC, Rothblum LI, Musch TI, Moore RL, Cheung JY (2000). Sprint training normalizes Ca2+ transients and SR function in postinfarction rat myocytes. J Appl Physiol.

[14] Jain M (2000). Angiotensin II receptor blockade attenuates the deleterious effects of exercise training on post-MI ventricular remodelling in rats. Cardiovasc Res.

[15] Brown DA, Jew KN, Sparagna GC, Musch TI, Moore RL (2003). Exercise training preserves coronary flow and reduces infarct size after ischemia-reperfusion in rat heart. J Appl Physiol.

[16] Hambrecht R (2000). Effects of exercise training on left ventricular function and peripheral resistance in patients with chronic heart failure. JAMA.

[17] Wisløff U (2002). Aerobic exercise reduces cardiomyocyte hypertrophy and increases contractility, Ca2+ sensitivity and SERCA-2 in rat after myocardial infarction. Cardiovasc Res.

[18] Barboza CA, Rocha LY, Mostarda CT, Figueroa D, Caperuto EC, De Angelis K, Irigoyen MC, Rodrigues B (2013). Ventricular and autonomic benefits of exercise training persist after detraining in infarcted rats. Eur J Appl Physiol.

[19] Grans CF, Feriani DJ, Abssamra MEV, Rocha LY, Carrozzi NM, Mostarda C, Figueroa DM, De Angelis K, Irigoyen MC, Rodrigues B (2014). Resistance training after myocardial infarction in rats: its role on cardiac and autonomic function. Arq Bras Cardiol.

[20] do Batista NE, Leite RD, Prestes J (2011). Câncer: benefícios do treinamento de força e aeróbio. Revista Da Educação Física/UEM.

[21] Ramires AT, Pretes J (2013). Treinamento de Força e Síndrome Metabólica: Uma Revisão Sistemática. Revista Brasileira de Cardiologia.

[22] da Silva EG, Dourado VZ (2008). Treinamento de Força Para Pacientes Com Doença Pulmonar Obstrutiva Crônica. Revista Brasileira de Medicina Do Esporte.

[23] Selig S, Carey M, Menzies D, Patterson J, Geerling R, Williams A, Bamroongsuk V, Toia D, KrumHare HD (2004). Moderate-intensity resistance exercise training in patients with chronic heart failure improves strength, endurance, heart rate variability, and forearm blood flow*1. J Cardiac Fail.

[24] Levinger I, Bronks R, Cody DV, Linton I, Davie A (2005). Resistance training for chronic heart failure patients on beta blocker medications. Int J Cardiol.

[25] Mandic S, Myers J, Selig SE, Levinger I (2012). Resistance versus aerobic exercise training in chronic heart failure. Curr Heart Fail Rep.

[26] Fontes-Carvalho R, Azevedo AI, Sampaio F, Teixeira M, Bettencourt N, Campos L, Gonçalves FR, Ribeiro VG, Azevedo A, Leite-Moreira A (2015). The effect of exercise training on diastolic and systolic function after acute myocardial infarction. Medicine.

[27] Grosse T, Kreulich T, Nägele H, Reer R, Petersen B, Braumann KM, Rödiger W (2001). Peripheral muscular strength training in patients with severe heart failure. Dtsch Z Sportmed.

[28] Pu CT, Johnson MT, Forman DE, Hausdorff JM, Roubenoff R, Foldvari M, Fielding RA, Fiatarone MA, Singh. (2001). Randomized trial of progressive resistance training to counteract the myopathy of chronic heart failure. J Appl Physiol.

[29] Tyni-Lenné R, Dencker K, Gordon A, Jansson E, Sylvén C (2001). Comprehensive local muscle training increases aerobic working capacity and quality of life and decreases neurohormonal activation in patients with chronic heart failure. Eur J Heart Fail.

[30] DS Ziaeddin, AH Far, K Azizbeigi. 2014. “Back to browse issues page effect of resistance and endurance training protocols on functional capacity and quality of life in male patients after myocardial infarction.” 3(1).

[31] Susan M, Oh PI, Thomas SG, Goodman JM (2008). Aerobic and resistance training in coronary disease. Med Sci Sports Exercise.

[32] Gayda M, Choquet D, Ahmaidi S (2009). Effects of exercise training modality on skeletal muscle fatigue in men with coronary heart disease. J Electromyogr Kinesiol.

[33] Karlsdottir AE, Foster C, Porcari JP, Palmer-McLean K, White-Kube R, Backes RC (2002). Hemodynamic responses during aerobic and resistance exercise. J Cardpulm Rehabil.

[34] Souza LM, Gomes MJ, Brandao BB, Pagan LU, Gatto M, Damatto FC, Rodrigues EA, Pontes THD, Borim PA, Fernandes AAH, Murata GM, Zornoff LAM, Azevedo PS, Okoshi K, Okoshi MP (2023). Effects of resistance exercise on slow-twitch soleus muscle of infarcted rats. Antioxidants.

[35] Werber-Zion G, Goldhammer E, Shaar A, Pollock ML (2004). Left ventricular function during strength testing and resistance exercise in patients with left ventricular dysfunction. J Cardpulm Rehabil.

[36] Senden PJ, Sabelis LW, Zonderland ML, Hulzebos EH, Bol E, Mosterd WL (2005). The effect of physical training on workload, upper leg muscle function and muscle areas in patients with chronic heart failure. Int J Cardiol.

[37] Hare DL, Ryan TM, Selig SE, Pellizzer A-M, Wrigley TV, Krum H (1999). Resistance exercise training increases muscle strength, endurance, and blood flow in patients with chronic heart failure. Am J Cardiol.

[38] Arthur HM, Gunn E, Thorpe KE, Ginis KM, Mataseje L, McCartney N, McKelvie RS (2007). Effect of aerobic vs combined aerobic-strength training on 1-Year, post-cardiac rehabilitation outcomes in women after a cardiac event. J Rehabil Med.

[39] Hung M-J (2004). Reduction of high-sensitivity C-reactive protein after treatment with anti-spastic agents in patients with coronary vasospastic angina and no hemodynamically significant coronary artery disease. Chest.

[40] Antonio EL, Dos Santos AA, Araujo SRR, Bocalini DS, dos Santos L, Fenelon G, Franco MF, Tucci PJF (2009). Left ventricle radio-frequency ablation in the rat: a new model of heart failure due to myocardial infarction homogeneous in size and low in mortality. J Cardiac Fail.

[41] Helber I, Dos Santos AA, Antonio EL, Flumignan RLG, Bocalini DS, Piccolo C, Gheorghiade M, Tucci PJF (2009). Digitoxin prolongs survival of female rats with heart failure due to large myocardial infarction. J Cardiac Fail.

[42] dos Santos L, Antonio EL, Souza AFM, Tucci PJF (2010). Use of afterload hemodynamic stress as a practical method for assessing cardiac performance in rats with heart failure. Can J Physiol Pharmacol.

[43] Dos Santos L, Mello AFS, Antonio EL, Tucci PJF (2008). Determination of myocardial infarction size in rats by echocardiography and tetrazolium staining: correlation, agreements, and simplifications. Braz J Med Biol Res.

[44] Duncan ND, Williams DA, Lynch GS (1998). Adaptations in rat skeletal muscle following long-term resistance exercise training. Eur J Appl Physiol.

[45] Hornberger TA, Farrar RP (2004). Physiological hypertrophy of the FHL muscle following 8 weeks of progressive resistance exercise in the rat. Can J Appl Physiol.

[46] Perilhão MS, Krause Neto W, da Silva AA, Alves LIS, Antonio EL, Medeiros A, Rica RL, Serra AJ, Tucci PJF, Bocalini DS (2020). Linear periodization of strength training in blocks attenuates hypertension and diastolic dysfunction with normalization of myocardial collagen content in spontaneously hypertensive rats. J Hypertens.

[47] Bocalini DS, Lima LS, de Andrade S, Madureira A, Rica RL, Nolasco R, dos Santos A, Serra J, Silva JA, Rodriguez D, Figueira A, Pontes FL (2012). Effects of circuit-based exercise programs on the body composition of elderly obese women. Clin Interv Aging.

[48] Bocalini DS, dos Santos L, Antonio EL, Santos AA, Davel AP, Rossoni LV, Vassalo DV, Tucci PJ (2012). Myocardial remodeling after large infarcts in rat converts post rest-potentiation in force decay. Arq Bras Cardiol.

[49] Rossoni LV, Xavier FE, Moreira CM, Falcochio D, Amanso AM, Tanoue CU, Carvalho CRO, Vassallo DV (2006). Ouabain-induced hypertension enhances left ventricular contractility in rats. Life Sci.

[50] dos Santos L, Gonçalves GA, Davel AP, Santos AA, Krieger JE, Rossoni LV, Tucci PJ (2013). Cell therapy prevents structural, functional and molecular remodeling of remote non-infarcted myocardium. Int J Cardiol.

[51] dos Santos L, Salles TA, Arruda-Junior DF, Campos LCG, Pereira AC, Ana LT, Antonio EL, Mansur AJ, Tucci PJF, Krieger JE, Girardi ACC (2013). Circulating dipeptidyl peptidase IV activity correlates with cardiac dysfunction in human and experimental heart failure. Circ Heart Fail.

[52] Gerdes AM, Liu Z, Zimmer HG (1994). Changes in nuclear size of cardiac myocytes during the development and progression of hypertrophy in rats. Cardioscience.

[53] Tucci PJF (2011). Características Fisiopatológicas Do Modelo de Insuficiência Cardíaca Pós-Infarto Do Miocárdio No Rato. Arq Bras Cardiol.

[54] Zornoff LAM, Paiva SAR, Minicucci MF, Spadaro J (2009). Infarto Do Miocárdio Experimental Em Ratos: Análise Do Modelo. Arq Bras Cardiol.

[55] Gao E, Lei YH, Xiying Shang Z, Huang M, Zuo L, Boucher M, Qian Fan J, Chuprun K, Ma XL, Koch WJ (2010). A novel and efficient model of coronary artery ligation and myocardial infarction in the mouse. Circ Res.

[56] Katsanos K, Mitsos S, Koletsis E, Bravou V, Karnabatidis D, Kolonitsiou F, Diamantopoulos A, Dougenis D, Siablis D (2012). Transauricular embolization of the rabbit coronary artery for experimental myocardial infarction: comparison of a minimally invasive closed-chest model with open-chest surgery. J Cardiothorac Surg.

[57] Kren L, Meluzin J, Pavlovsky Z, Mayer J, Kala P, Groch L, Hornacek I, Rauser P, Vlasin M (2010). Experimental model of myocardial infarction: histopathology and reperfusion damage revisited. Pathol Res Pract.

[58] Heimburger RF (1946). Injection into pericardial sac and ligation of coronary artery of the rat. Arch Surg (1920).

[59] Fishbein MC, Maclean D, Maroko PR (1978). The histopathologic evolution of myocardial infarction. Chest.

[60] Litwin SE (1995). “The rat model of postinfarction. Heart Failure.

[61] Freimann S, Kessler-Icekson G, Shahar I, Radom-Aizik S, Yitzhaky A, Eldar M, Scheinowitz M (2009). Exercise training alters the molecular response to myocardial infarction. Med Sci Sports Exerc.

[62] Gomes MJ, Pagan LU, Lima ARR, Reyes DRA, Martinez PF, Damatto FC, Pontes THD, Rodrigues EA, Souza LM, Tosta IF, Fernandes AAH, Zornoff LAM, Okoshi K, Okoshi MP (2020). Effects of aerobic and resistance exercise on cardiac remodelling and skeletal muscle oxidative stress of infarcted rats. J Cell Mol Med.

[63] Zhang X-Q, Musch TI, Zelis R, Cheung JY (1999). Effects of impaired Ca ^2+^ homeostasis on contraction in postinfarction myocytes. J Appl Physiol.

[64] Sofia RR, Serra AJ, Antonio EL, Manchini MT, Alves FA, de Oliveira V, Teixeira PC, Tucci PJF (2014). Gender-based differences in cardiac remodeling and ILK expression after myocardial infarction. Arq Bras Cardiol.

[65] Hannigan GE, Coles JG, Dedhar S (2007). Integrin-linked kinase at the heart of cardiac contractility, repair, and disease. Circ Res.

[66] Ding L, Dong Li, Chen X, Zhang L, Xiaoyu Xu, Ferro A, Biao Xu (2009). Increased expression of integrin-linked kinase attenuates left ventricular remodeling and improves cardiac function after myocardial infarction. Circulation.

[67] Hernando V, Inserte J, Sartório CL, Parra VM, Poncelas-Nozal M, Garcia-Dorado D (2010). Calpain translocation and activation as pharmacological targets during myocardial ischemia/reperfusion. J Mol Cell Cardiol.

[68] Kannan MM, Darlin Quine S (2013). Ellagic acid inhibits cardiac arrhythmias, hypertrophy and hyperlipidaemia during myocardial infarction in rats. Metabolism.

[69] Aikawa Y, Wakasugi Y, Narukawa T, Yamashita T, Sasai N, Umemura Y, Omi N, Ohtsuki M (2019). Jump exercise and food restriction on bone parameters in young female rats. Calcif Tissue Int.

[70] Okubo R, Sanada LS, Castania VA, Louzada MJQ, de Paula FJA, Maffulli N, Shimano AC (2017). Jumping exercise preserves bone mineral density and mechanical properties in osteopenic ovariectomized rats even following established osteopenia. Osteoporosis Int.

[71] Ahmadiasl N, Najafipour H, Soufi FG, Jafari A (2012). Effect of short- and long-term strength exercise on cardiac oxidative stress and performance in rat. J Physiol Biochem.

[72] Barauna VG, Rosa KT, Irigoyen MC, de Oliveira EM (2007). Effects of resistance training on ventricular function and hypertrophy in a rat model. Clin Med Res.

[73] Garza MA, Wason EA, Cruger JR, Chung E, Zhang JQ (2019). Strength training attenuates post-infarct cardiac dysfunction and remodeling. J Physiol Sci.

[74] Walter KN, de Assis W, Silva TV, de Oliveira A, Dos SantosVilasBoas AE, Ciena AP, Anaruma CA, Gorzi A, Caperuto ÉC, Gama EF (2022). Muscle hypertrophy is correlated with load progression delta, climb volume, and total load volume in rodents undergoing different ladder-based resistance training protocols. Tissue Cell.

[75] Cassilhas RC, Reis IT, Venâncio D, Fernandes J, Tufik S, Túliode Mello M (2013). Animal model for progressive resistance exercise: a detailed description of model and its implications for basic research in exercise. Motriz Revista de Educação Física.

[76] Nascimento V, Neto WK, Gonçalves L, Maifrino LBM, Souza RR, Gama EF (2013). Morphoquantitative analysis revealed triceps brachialis muscle hypertrophy by specific resistance training equipment in rats. J Morphol Sci.

[77] Walter KN, Adriano C, Carlos A, Eliane G, Wellington S (2016). Vertical climbing for rodent resistance training: a discussion about training parameters. Int J Sports Sci.

[78] Lourenço Í, Krause Neto W, Amorim LDSP (2020). Muscle hypertrophy and ladder-based resistance training for rodents: a systematic review and meta-analysis. Physiol Rep.

[79] Shimojo GL, Palma RK, Brito JO, Sanches IC, Irigoyen MC, De Angelis K (2015). Dynamic resistance training decreases sympathetic tone in hypertensive ovariectomized rats. Braz J Med Biol Res.

[80] Hwang C-L, Chien C-L, Ying-Tai Wu (2010). Resistance training increases 6-minute walk distance in people with chronic heart failure: a systematic review. J Physiother.

[81] Neto WK, de Assis Silva W, Ciena AP, Bocalini D, Nucci RAB, Anaruma CA, Gama EF (2018). Total training load may explain similar strength gains and muscle hypertrophy seen in aged rats submitted to resistance training and anabolic steroids. Aging Male Off J Int Soc Study Aging Male.

[82] Portes LA, Tucci PJF (2006). O treinamento Físico Por Natação atenua o remodelamento miocárdico e congestão pulmonar em ratas wistar com insuficiência cardíaca secundária a infarto do miocárdio. Arq Bras Cardiol.

[83] de Campos Neitzke WS, de Macedo RM, Francisco JC, Santos PC, Lopes APS, de Meira LF, de Carvalho KAT, Neto JRF, de Macedo ACB, Guarita-Souza LC (2018). Impact of a high-intensity training on ventricular function in rats after acute myocardial infarction. Arq Bras Cardiol.

[84] Andreollo NA, Freitas E, dos Santos M, Araújo R, Lopes LR (2012). Idade Dos Ratos versus Idade Humana: Qual é a Relação?. ABCD Arquivos Brasileiros de Cirurgia Digestiva (São Paulo).

[85] Maia CB, Mengal V, Brasil GA, Peluso AA, Treebak JT, Endlich PW, de Almeida SA, de Abreu GR (2022). Ellagic acid prevents myocardial infarction-induced left ventricular diastolic dysfunction in ovariectomized rats. J Nutritional Biochem.

[86] Couto GK, Luiz RG, Britto JGM, Rossoni LV (2015). Enhanced nitric oxide bioavailability in coronary arteries prevents the onset of heart failure in rats with myocardial infarction. J Mol Cell Cardiol.

[87] Feriani DJ, Coelho-Júnior HJ, Irigoyen MC, Rodrigues B (2018). Protective effects of accumulated aerobic exercise in infarcted old rats. Int J Cardiovascular Sci.

[88] Hentschke VS, Capalonga L, Rossato DD, Perini JL, Alves JP, Quagliotto E, Stefani GP, Karsten M, Pontes M, Dal Lago P (2017). Functional capacity in a rat model of heart failure: impact of myocardial infarct size. Exp Physiol.

[89] Picollo CT, dos Santos AA, Antonio EL, Silva JMA, Bocalini D, Serra AJ, Ihara SSM, Tucci PJF (2020). Digitoxin attenuates heart failure, reduces myocardial hypertrophy, and preserves the calcium-binding proteins in infarcted rats. J Cardiovasc Pharmacol Ther.

[90] Moreira FL, da Silva GW (2022). Não Tratamos a Congestão Pulmonar e Sistêmica Na Insuficiência Cardíaca Aguda Adequadamente. JBMEDE J Brasileiro de Med de Emergência.

[91] Swynghedauw B (1999). Molecular mechanisms of myocardial remodeling. Physiol Rev.

[92] Gosselin LE (2000). Attenuation of force deficit after lengthening contractions in soleus muscle from trained rats. J Appl Physiol.

[93] Min JY, Sandmann S, Meissner A, Unger T, Simon R (1999). Differential effects of mibefradil, verapamil, and amlodipine on myocardial function and intracellular Ca(2+) handling in rats with chronic myocardial infarction. J Pharmacol Exp Ther.

[94] Min J-Y, Yang Y, Converso KL, Liu L, Huang Q, Morgan JP, Xiao Y-F (2002). Transplantation of embryonic stem cells improves cardiac function in postinfarcted rats. J Appl Physiol.

[95] Sjaastad I, Birkeland JA, Ferrier G, Howlett S, Skomedal T, Bjornerheim R, Wasserstrom JA, Sejersted OM (2005). Defective excitation-contraction coupling in hearts of rats with congestive heart failure. Acta Physiol Scand.

[96] Wasserstrom J, Andrew EH, Sjaastad I, Lunde PK, Ødegaard A, Sejersted OM (2000). Altered E-C coupling in rat ventricular myocytes from failing hearts 6 Wk after MI. Am J Physiol-Heart Circulatory Physiol.

[97] Zimmerman SD, Paul Thomas D, Velleman SG, Li X, Hansen TR, McCormick RJ (2001). Time course of collagen and decorin changes in rat cardiac and skeletal muscle post-MI. Am J Physiol-Heart Circulatory Physiol.

[98] Azevedo PS, Polegato BF, Minicucci MF, Paiva SAR, Zornoff LAM (2016). Cardiac remodeling: concepts, clinical impact, pathophysiological mechanisms and pharmacologic treatment. Arq Bras Cardiol.

[99] Liao Z, Li D, Chen Y, Li Y, Huang R, Zhu K, Chen H, Yuan Z, Zheng X, Zhao H, Qin Pu, Qi X, Cai D (2019). Early moderate exercise benefits myocardial infarction healing via improvement of inflammation and ventricular remodelling in rats. J Cell Mol Med.

[100] Pereira AJ, Nunes RB, da CunhaFerreira D, Stefani GP, Jaenisch RB, Dal Lago PD (2017). High-intensity resistance training alone or combined with aerobic training improves strength, heart function and collagen in rats with heart failure. Am J Trans Res.

[101] Reza G, Gaeini A, Kordi MR, Aboutaleb N, Afousi AG (2018). Effect of one period of high-intensity interval training on myocardial collagen-1 and TGF-Î^2^1 and cardiac function in post ischemia-reperfusion rats. Daneshvar Med Basic Clin Res J.

[102] Bocalini DS, Beutel A, Bergamaschi CT, Tucci PJ, Campos RR (2014). Treadmill exercise training prevents myocardial mechanical dysfunction induced by androgenic-anabolic steroid treatment in rats. PLoS ONE.

[103] Bocalini DS, Tucci PJF (2009). Developed force of papillary muscle what index correctly indicates contractile capacity?. Int Heart J.

[104] Chen J, Feller GM, Barbato JC, Periyasamy S, Xie Z-J, Koch LG, Shapiro JI, Britton SL (2001). Cardiac performance in inbred rat genetic models of low and high running capacity. J Physiol.

[105] Song W, Vikhorev PG, Kashyap MN, Rowlands C, Ferenczi MA, Woledge RC, MacLeod K, Marston S, Curtin NA (2013). Mechanical and energetic properties of papillary muscle from *ACTC* E99K transgenic mouse models of hypertrophic cardiomyopathy. Am J Physiol-Heart Circulatory Physiol.

[106] Veiga EC, de Arruda L, Portes A, Bocalini DS, Antonio EL, Alberta A, dos Santos M, Santos H, Silva FA, Tucci PJF (2013). Repercussões Cardíacas Após Infarto Do Miocárdio Em Ratas Submetidas Previamente a Exercício Físico. Arq Bras Cardiol.

[107] Sjaastad I, Sejersted OM, Ilebekk A, Bjørnerheim R (2000). Echocardiographic criteria for detection of postinfarction congestive heart failure in rats. J Appl Physiol.

[108] Igawa A, Nozawa T, Yoshida N, Fujii N, Kato B-I, Inoue M, Tazawa S, Yamada Y, Asanoi H, Inoue H (2002). Effects of the angiotensin-converting enzyme inhibitor enalapril on sympathetic neuronal function and BETA.-adrenergic desensitization in heart failure after myocardial infarction in rats. Jpn Heart J.

[109] Sandmann S, Spitznagel H, Chung O, Xia Q-G, Illner S, Jänichen G, Rossius B, Daemen MJAP, Unger T (1998). Effects of the calcium channel antagonist mibefradil on haemodynamic and morphological parameters in myocardial infarction-induced cardiac failure in rats. Cardiovasc Res.

[110] Litwin SE, Litwin CM, Raya TE, Warner AL, Goldman S (1991). Contractility and stiffness of noninfarcted myocardium after coronary ligation in rats. Effects of chronic angiotensin converting enzyme inhibition. Circulation.

[111] Qi X-L, Stewart DJ, Gosselin H, Azad A, Picard P, Andries L, Sys SU, Brutsaert DL, Rouleau JL (1999). Improvement of endocardial and vascular endothelial function on myocardial performance by captopril treatment in postinfarct rat hearts. Circulation.

[112] Cokkinos D, Belogianneas C (2016). Left ventricular remodelling: a problem in search of solutions. Eur Cardiol Rev.

[113] He J (2001). Reduction in Density of transverse tubules and L-type Ca2+ channels in canine tachycardia-induced heart failure. Cardiovasc Res.

[114] Mukherjee R, Hewett KW, Walker JD, Basler CG, Spinale FG (1998). Changes in L-type calcium channel abundance and function during the transition to pacing-induced congestive heart failure. Cardiovasc Res.

[115] Mukherjee R, Spinale FG (1998). L-type calcium channel abundance and function with cardiac hypertrophy and failure: a review. J Mol Cell Cardiol.

[116] UM Ravens, IM Noble, WA SEED. 1992. *The Interval-Force Relationship of the Heart*. New York.

[117] Ravens U, Link S, Gath J, Noble MIM (1995). Post-rest potentiation and its decay after inotropic interventions in isolated rat heart muscle. Pharmacol Toxicol.

[118] Endoh M (2004). Force–frequency relationship in intact mammalian ventricular myocardium: physiological and pathophysiological relevance. Eur J Pharmacol.

[119] Kemi OJ, Haram PM, Loennechen JP, Osnes JB, Skomedal T, Wisløff U, Ellingsen Ø (2005). Moderate vs. high exercise intensity: differential effects on aerobic fitness, cardiomyocyte contractility, and endothelial function. Cardiovasc Res.

[120] Kemi OJ, Ceci M, Condorelli G, Smith GL, Wisloff U (2008). Myocardial sarcoplasmic reticulum Ca2+ ATPase function is increased by aerobic interval training. Eur J Cardiovascular Prev Rehabil.

